# Is delirium a specific complication of viral acute respiratory distress syndrome?

**DOI:** 10.1186/s13054-020-03136-6

**Published:** 2020-07-09

**Authors:** Markus Jäckel, Xavier Bemtgen, Tobias Wengenmayer, Christoph Bode, Paul Marc Biever, Dawid Leander Staudacher

**Affiliations:** 1 Department of Cardiology and Angiology I, Faculty of Medicine, Heart Center Freiburg University, University of Freiburg, Hugstetter Strasse 55, 79106 Freiburg, Germany; 2 Department of Medicine III (Interdisciplinary Medical Intensive Care), Medical Center, Faculty of Medicine, University of Freiburg, Freiburg, Germany

**Keywords:** ARDS, Delirium, Intensive care unit, NuDesc, SARS-CoV2

Acute respiratory distress syndrome (ARDS) caused by the novel coronavirus SARS-CoV-2 is associated with a high rate of delirium resulting in encephalopathy, prominent agitation, and confusion [1]. Considering neurotropism of coronaviruses, a direct central nervous system invasion resulting in encephalopathy of SARS-CoV2 is discussed [2, 3]. Recent data reported an enhancement in leptomeningeal spaces and bilateral frontotemporal hypoperfusion in SARS-CoV-2 [1]. Since delirium however might also be caused by the systemic injury in critical illness [4], it remains debatable if the high rate of delirium is specifically associated with SARS-CoV-2 or rather a common complication of viral ARDS. We therefore compared delirium in ARDS patients caused by either SARS-CoV-2 or influenza A and B viruses.

We performed a single-center retrospective register analysis including invasive ventilated patients with ARDS and SARS-Cov-2 or influenza infection treated between 2015 and May 2020. We analyzed delirium by NuDesc (nursing delirium screening scale) score and RASS (Richmond agitation and sedation scale) score which are routinely assessed three times a day by especially trained nurses in all patients on our ICU. The NuDesc score is approved and shows a high sensitivity and specifity [5].

A total of 83 patients with ARDS were identified (44 and 39; with SARS-Cov-2 and influenza, respectively). Thirty-seven (22 and 15) died before extubation and 10 (2 and 8) were transferred with tracheotomia without the possibility of delirium evaluation using a verbal test. We therefore analyzed 36 (20 and 16) patients. Besides of age (patients with SARS-Cov-2 infection were significantly older), groups were homogenous (see Table 1).

**Table 1:** Characteristics of patients with ARDS caused by SARS-CoV-2 or influenza A/B. For laboratory data, maximum values are shown. *p* value reported in bold if difference is significant (*p* < 0.05). Data are given as mean ± standard deviation or number of patients (percent of all patients in group). ^a^Student’s *t* test; ^b^Welch *t* test, ^c^chi-square test; ^d^Fisher’s exact test

	Influenza (***N*** = 16)	COVID-19 (***N*** = 20)	*p*
Age	54.31 ± 12.36	65.48 ± 10.99	**0.007**^a^
Female	5 (31.3%)	4 (20.0%)	0.470^d^
ICU stay (days)	19.85 ± 12.09	21.05 ± 11.77	0.765^a^
Death	0 (0%)	2 (10.0%)	0.492^d^
Severe ARDS	11 (68.8%)	9 (45.0%)	0.154^c^
Days of invasive ventilation	18.28 ± 15.61	15.47 ± 10.34	0.522^a^
TISS 10	16.63 ± 5.73	15.25 ± 6.77	0.521^a^
SAPS 2	40.38 ± 9.88	44.70 ± 11.13	0.232
Noradrenalin > 1 mg/h	8 (50.0%)	10 (50%)	1.000
Renal replacement therapy	4 (25.0%)	6 (30.0%)	1.000
Lactat mmol/l	3.35 ± 1.82	3.07 ± 2.23	0.369
CRP mg/dl	302.99 ± 96.89	257.34 ± 84.46	0.140
Procalcitonin ng/ml	59.22 ± 106.43	17.19 ± 33.46	0.159
Delirium	12 (75.0%)	13 (65.0%)	0.718
Delirium duration (days)	2.83 ± 2.44	5.08 ± 4.29	0.126
NuDesc score at maximum	3.67 ± 1.78	5.15 ± 2.58	0.109
Delirium onset after extubation (days)	0.80 ± 1.55	0.50 ± 1.08	0.622

Of all analyzed patients 69.4% (65.0 and 75.0% with SARS-CoV-2 and influenza, respectively) were diagnosed with delirium at any time during the ICU stay. Delirium duration tended to be longer in patients with SARS-CoV-2 (5.1 ± 4.3 days vs. 2.8 ± 2.4 days, *p* = 0.13). Delirium severity, defined as maximum of NuDesc score, also tended to be more distinctive in SARS-Cov-2 patients (NuDesc score at maximum: 5.2 ± 2.6 vs. 3.7 ± 1.8, *p* = 0.11). The onset of delirium after extubation was similar (0.50 ± 1.08 days vs. 0.8 ± 1.6 days). For the delirium presentation, see Fig. 1.

**Fig. 1:**
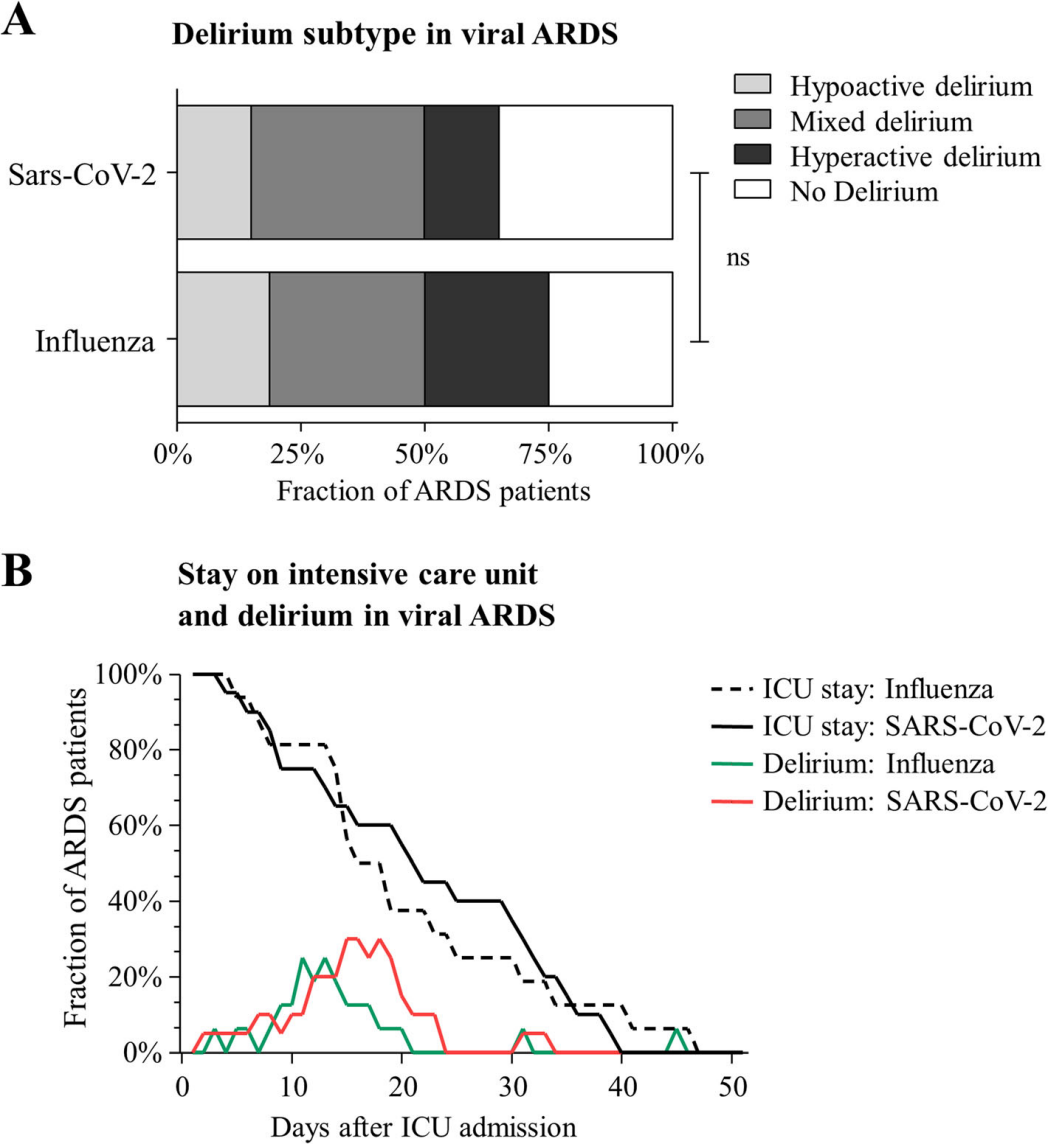
Delirium presentation and duration. Graph shows delirium distribution of hyperactive/hypoactive/mixed delirium and no delirium shown in percent in patients with ARDS caused by SARS-CoV-2 or influenza A/B (**a**). Graph shows stay on the intensive care unit and fraction of delirium positive patients shown in percent in patients with ARDS caused by SARS-CoV-2 or influenza A/B (**b**)

In this registry study of delirium in viral ARDS, we found no statistical significant difference in delirium prevalence, intensity, or type of delirium comparing patients with SARS-CoV-2 to those with influenza. We therefore hypothesize that delirium observed in COVID-19 patients has to be considered a complication of ARDS rather than SARS-CoV-2 specific. Considering the retrospective nature of data presented here, our results have to be considered hypothesis generating and have to be confirmed in a larger patient collective.

## Data Availability

The datasets used and analyzed during the current study are available from the corresponding author on reasonable request.

## Abbreviations

ARDS: Acute respiratory distress syndrome
NuDesc: Nursing delirium screening scale
RASS: Richmond agitation and sedation scale
TISS: Therapeutic Intervention Scoring System
SAPS: Simplified Acute Physiology Score
